# Penile Gangrene with Abscess Formation after Modified Al-Ghorab Shunt for Idiopathic Ischemic Priapism

**DOI:** 10.1155/2014/705417

**Published:** 2014-09-08

**Authors:** Beneranda S. Ford-Glanton, Parth Patel, Sameer Siddiqui

**Affiliations:** ^1^Division of Urology, St. Louis University Hospital, St. Louis, MO 63103, USA; ^2^St. Louis University School of Medicine, St. Louis, MO 63103, USA

## Abstract

Penile gangrene is a rare but unfortunate complication of surgical intervention and priapism shunts. The literature regarding penile gangrene following surgical correction of priapism is sparse, the majority of which dates back to thirty to forty years. Here, we present the case of a 60-year-old man who presented with priapism that required operative management with a modified Al-Ghorab shunt and eventually suffered from complete necrosis of the penis with abscess formation in both corpora cavernosa.

## 1. Introduction

Penile gangrene is an uncommon and unfortunate disease process. It is a known sequela of severe systemic vascular disease and a rare consequence of surgical intervention for hypospadias repair and priapism shunts [1]. There is little mention of this complication following surgical correction of priapism in the recent literature, as the majority of literature on postoperative penile gangrene dates back to the 1970s and 1980s [2]. More specifically, a case of complete abscess formation within both corpora cavernosa is yet to be reported in English literature, making this a unique case that merits documentation.

## 2. Case Report

A 60-year-old male presented to an outside hospital after three days of painful and prolonged erection. The patient has a history of non-insulin-dependent diabetes mellitus, hypertension, hepatitis C, and hyperlipidemia; he also smokes 1.5 packs of tobacco per day. The outside urologist was only able to achieve partial detumescence with a phenylephrine injection, but the patient was discharged home from the emergency department. He presented the next day to our facility with continued painful erection and reported that the erection never completely resolved from the previous day. The patient was noted to have an elevated temperature (103°F) and a WBC count of 27,200/*μ*L. We attempted phenylephrine and saline irrigation but observed only a temporary response. The patient received cefoxitin following the intracorporal phenylephrine injections, in anticipation of possibly undergoing a distal shunt procedure in the operating room. Because the patient presented with site-specific pain, fever, and an elevated WBC count, it was deemed that surgery was the best management option at that point to reduce the risk of sepsis. The patient underwent a modified Al-Ghorab shunt procedure. Postoperatively, the patient had an uncomplicated course and was discharged home with a 10-day course of cephalexin.

**Figure 1:**
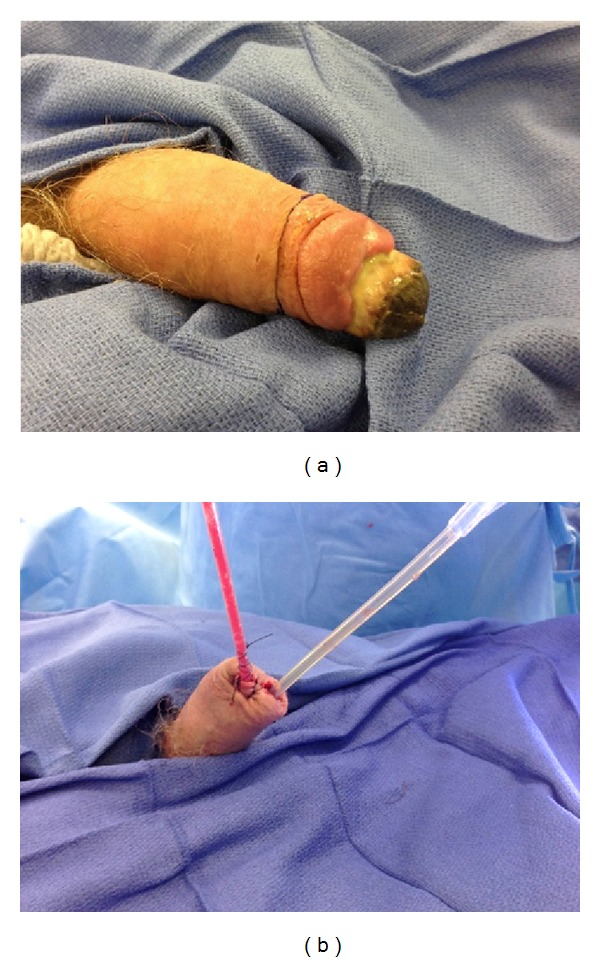
Gross images prior to and following distal penectomy for gangrene.

The patient presented to our clinic on postoperative day 21 and was noted to have thick eschar on the glans penis. At the time, the wound was deemed to heal appropriately. Approximately one month later, the patient was seen in clinic again with complete necrosis of the glans penis, as well as purulent discharge, but the patient was voiding without difficulty. Magnetic resonance imaging demonstrated a 14 cm abscess in both of the corpora cavernosa. The patient was taken urgently to the operating room for distal penectomy with irrigation of the corpora and drain placement; the urethra was largely preserved (Figure 1). The patient's wound culture was positive for* Staphylococcus aureus* and he was treated with piperacillin + tazobactam and vancomycin while being an inpatient and with clindamycin as an outpatient. He was discharged home with both a Foley catheter and a Jackson-Pratt drain.

## 3. Discussion and Analysis

Penile ischemia after surgical intervention for priapism was primarily described when compression dressings were frequently used in the immediate postoperative period [3]. Once urologists learned that compression therapy was increasing the rate of penile ischemia and gangrene, the practice has since been halted, but iatrogenic injury remains the primary cause of penile gangrene today [1]. Since the early 1980s, there have only been a handful of cases in the literature of penile gangrene after surgery for priapism [4].

The vascular supply to the penis is robust and is supplied by three branches of internal pudendal artery: the dorsal artery, the cavernosal artery, and the bulbourethral artery. Penile gangrene is rarely encountered because of this rich collateral circulation of penis. However, the end-organ vasculature of the penile cavernous tree is susceptible to arterial insufficiency caused by a systemic vasculopathic state [5, 6]. According to the literature, risk factors for penile gangrene include renal insufficiency, diabetes mellitus, thromboemboli, coagulopathy, operation, ligation of penile circulation, peripheral vascular disease, and Kaposi's sarcoma [7–10].

The case may be that patients who end up with penile gangrene have unrecognized long term arterial insufficiency and any trauma-induced local inflammation could increase the requirement of oxygen and nutrients. This would impose further burden on the already impaired penile vascular supply and result in penile ischemia. Our patient did report a history of erectile dysfunction, which indicates probable small vessel disease. In reviewing the literature, the largest case series postulated that infection, preceded by fever, was the key factor in developing gangrene requiring amputation [1]. Our patient's elevated temperature and prior history of intracavernosal phenylephrine injections also increase the risk of infection. Although the patient received perioperative antibiotic prophylaxis, the infectious process may have already overwhelmed a compromised vasculature system.

While surgery for priapism is a common and accepted form of treatment, postsurgical penile necrosis and gangrene or the prevention thereof are not well described. While a review of the literature does not elicit any new information on how to prevent this complication, it does remind us to remain vigilant in monitoring these patients for the signs of penile ischemia, because preservation of penile tissue requires prevention or early intervention. Prevention should focus on pre- and postprocedure antibiotic prophylaxis for any intervention, whether it is simple bedside irrigation of the corpora or shunt placement in the operating room. Minimizing the risk of infection should also include attempts to manage priapism conservatively and, if operative management cannot be avoided, an effort to reduce disturbance of the penile blood supply. Treatment for penile gangrene can include conservative therapy such as debridement, but purulent abscess formation and necrotic glans penis may leave partial penectomy as the best therapeutic option, as was the case in our patient.

## 4. Conclusion

Penile gangrene is a rare and devastating complication. The process is irreversible and must be recognized early in order to prevent further tissue loss. As urologists, we must maintain a high index of suspicion and be prepared to act quickly to preserve penile tissue and length.
